# Comparison of hepatitis B and SARS-CoV2 vaccination rates in people who attended Drugs and Addiction Centres

**DOI:** 10.3389/fpubh.2023.1258095

**Published:** 2024-01-16

**Authors:** Diana Corona-Mata, Antonio Rivero-Juárez, Ángela Camacho, Laura Ruiz-Torres, Inmaculada Ruiz-Cáceres, Ana Belén Pérez, Bartolomé de la Fuente Darder, David Cáceres-Anillo, María de Guía Castro-Granados, María Lizaur-Barbudo, María Victoria Cabrera-Gisbert, Justa Redondo-Écija, Ana Aparicio-Aparicio, Leticia Manchado-López, Luciano Cobos, Ignacio Pérez-Valero, Antonio Rivero

**Affiliations:** ^1^Infectious Diseases Department, Maimonides Institute of Biomedical Research of Córdoba (IMIBIC), Reina Sofía University Hospital of Córdoba, University of Córdoba, Córdoba, Spain; ^2^CIBERINFEC, ISCIII – CIBER of Infectious Diseases, Instituto de Salud Carlos III, Madrid, Spain; ^3^Microbiology Unit, Reina Sofía University Hospital of Córdoba, Maimonides Institute of Biomedical Research of Córdoba (IMIBIC), Córdoba, Spain; ^4^Unidad de Drogas y Adicciones-CPD (UDA-CPD), Instituto Provincial Bienestar Social, Diputación Córdoba, Córdoba, Spain; ^5^Renacer Home, Córdoba, Spain

**Keywords:** hepatitis B virus, COVID-19 disease, SARS-CoV-2, drugs addiction, vaccination, prevention

## Abstract

**Background and aims:**

Persons with substance use disorder are at increased risk for hepatitis B virus (HBV) infection. Although most of them are attached to social health centers, the vaccination rate in this group is low. In this context, we designed a study to evaluate the prevalence of users of drug addiction centers (DAC) not immunized against hepatitis B and to compare the rate of vaccination against hepatitis B with the rate of immunization against SARS-Cov-2 in 2 years of follow-up.

**Design:**

Retrospective study that included individuals attended at DAC. Patients were screened at baseline (June 2020–January 2021) for HBV immunization. Individuals with HBsAb < 10 IU/mL were recommended to receive hepatitis B vaccine, during follow-up (January 2021–October 2022). At the end of follow-up, the HBV vaccination rate among candidates was determined and compared with the vaccination rate against SARS-Cov-2 in this population in the same period.

**Findings:**

A total of 325 subjects were surveyed and tested. At baseline, the 65% (211/325) of were candidates to initiate vaccination and were advisor to HBV vaccination. During the follow-up 15 individuals received at least one dose of HBV vaccine, supposing a vaccination rate of 7.2%. In the same period, 186 individuals received at least one dose against SARS-Cov-2, representing a vaccination rate of 83%. The comparison between vaccination rates reached statistically significant (*p* < 0.001).

**Conclusion:**

Our study manifests a low rate of immunization against HBV in DAC users, despite a high level of immunization for SARS-Cov-2 during the same period in the same population. Consequently, the lack of immunization against HVB in this population might be related with health policy issue more than to individuals linked to care and awareness. A similar approach for vaccination intended for SARS-CoV2 should be applied in high-risk population to warrant the success of immunization program against other preventable diseases such as HBV.

## Graphical abstract

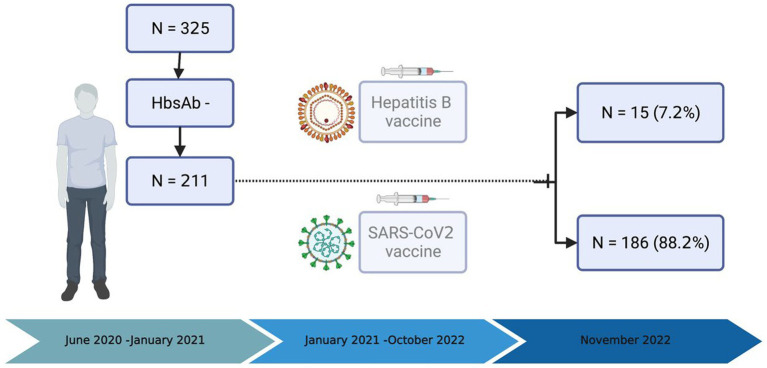

## Introduction

Hepatitis B infection is a major cause of liver disease worldwide. WHO estimates that 296 million people were living with chronic hepatitis B virus (HBV) infection in 2019, with 1.5 million new infections occurring each year. Moreover, the problem is aggravated by the high rates of underdiagnosis of this disease. It is estimated that only 10% of the infected population has been diagnosed (1). HBV infection can progress to chronicity and develop liver cirrhosis and complications, ranked as a major cause of end-stage liver disease and death (2). Although there are drugs capable of inhibiting viral replication, HBV is considered an infectious disease with a high risk of chronicity, and vaccination is the only way to prevent its clinical impact. Since 1981, vaccination strategies have been implemented in groups at risk of infection and later extended to the general population (3). WHO recommends vaccination schedules at birth and in early childhood. Thus, it is estimated that 80% of the world’s population reaches a vaccination coverage with 3 doses against hepatitis B (4). The contribution of hepatitis B vaccination in preventing infection and reducing hidden hepatitis B has been massive (5).

Despite the availability of an effective vaccine, the prevalence of hepatitis B infection remains high, in part due to hidden infection, late diagnosis, difficulty in accessing treatment, and non-immunization in certain groups of individuals at higher risk of hepatitis B infection (6). People with substance use disorders and people linked to Drugs and Addiction Centers (DACs) constitute a high-risk group for acquiring and spreading HBV infection or have severe complications and therefore may establish a major barrier to the WHO’s 2030 goal of elimination of the disease (7) especially those who inject drugs. A recent meta-analysis found that 15.6 million people in 2015 were injecting drugs throughout the world. Of them, 9% (5.1%–13.2%) suffer from chronic HBV infection (8). Identifying HBV unimmunized DAC users and achieving high vaccination rates in them is an essential strategy to eliminate hepatitis B. It is known that access to this population, follow-up and treatment is complicated, so vaccination rates are low. Some studies show low rates of complete hepatitis B vaccination in individuals attending drug dependence centers, especially in younger populations or those with longer vaccination schedules (6 month regimens) (9). Ensuring user adherence to the health system and fostering an understanding of the importance of preventing infections, such as hepatitis B, can be pivotal. However, the health system plays a critical role by shouldering the responsibility of providing adequate information and explaining the risks associated with not achieving immunization. Vaccination compliance is a fundamental milestone in the healthcare system. This example can be illustrated by the SARS-Cov-2 vaccination strategy.

Mass vaccination of the population against SARS-Cov-2 was a decisive tool for the control of the pandemic in the world. This was due to the fact that the information campaigns managed to arouse a high level of interest in being vaccinated in a large part of the population. Moreover, the high impact on mortality of SARS-Cov-2 and the confidence placed in obtaining an effective vaccine for the prevention of a disease that had an outbreak and repercussions on the lives of everyone had an added value for the acceptance of people to follow the preventive measures recommended by the health authorities and get vaccinated. In this scenario, we propose to evaluate the vaccination initiation rates against Hepatitis B in DAC users and compare them with the vaccination rates obtained against SARS-Cov-2 in this same population.

## Materials and methods

### Study design and population

Retrospective analysis of a study designed to screen and diagnose viral hepatitis in individuals linked to Drugs and Addiction Centres (DAC) from Cordoba (Southern Spain). This analysis aims to assess hepatitis B vaccination rates based on the standard strategy recommended for vaccination, and to compare them with the SARS-CoV-2 rates obtained through the intensive strategy involving health authorities’ intervention from June 2020 to November 2022.

The study subjects had previously participated in a study evaluating a comprehensive strategy for detecting and treating hepatitis C infection in Cordoba (10). The healthy volunteers were included in the present study.

At baseline, individuals were screened for hepatitis B serology between June 2020 and January 2021. This screening includes hepatitis B surface antigen tests, total hepatitis B surface antibody, and hepatitis B core antibody. Patients without HBV immunity (defined as anti-HBsAb antibody titer < 10 IU/mL) were referred to reference DAC with the recommendation of HBV vaccination.

Individuals at risk (not immunized to hepatitis B) were recommended to receive vaccination against HVB from baseline up to November 2022 (end of the study). The Infectious Diseases Unit informed the participant of the need to be vaccinated against HBV and recommended to request an appointment at their reference center to complete the vaccination. In our country, vaccination against HBV in adults is covered by the National and Regional Health System, free of charge, in those users at risk of immunosuppression, high risk of infection and dissemination, occupational risk or comorbidities with risk of chronic liver disease with a vaccination schedule of three doses, administered in month 0, 2, and 6 from the prescription (11).

Simultaneously, due to the global pandemic situation of the disease, the Health Authorities supported by the National Government carried out an intensive and universal vaccination strategy against SARS-CoV2. The general population was stratified and prioritized according to risk factors for severe COVID-19 infection and vaccinated in a staggered manner from the availability of the first vaccines in January 2021. The vaccination points were managed by the Preventive and Epidemiology Services, coordinated with the Primary Care Health Districts. Special vaccination teams were created, composed mostly of nursing staff, who, through municipal and health censuses, prioritized the population according to government guidelines. Vaccination was free of charge (12). Adults belonging to this cohort were summoned by census at health centers and vaccination points since June 2021.

The Public Andalusian Health system has a single, universal, and digitized registry of users’ medical records, as well as of the vaccines administered. All the health data of individuals are collected in it, and health providers have access to it to afford the best possible health care. To assess the acceptance of this population to receive vaccines, we compared the rate of hepatitis B vaccination with the rate of vaccination against SARS-CoV-2 in the same period.

### Variables and definition

The primary endpoint of the study was the initiation of HBV vaccination, defined as receiving at least one dose of the vaccine during the study period in candidate individuals. We decided to assess vaccination acceptance by reviewing their electronic vaccination records. The objective was to know how many had complied with the recommendation to vaccinate after counseling. This measure was objective for all and could be assessed by analyzing the vaccination records. Baseline variables including age (categorized into upper or lower 40 years), gender and history of drug use were registered. Patients with a history of injection drug use were on Opioid Substitute Therapy (OST).

### Statistical analysis

A comprehensive examination of the data involved a descriptive analysis, wherein categorical variables were depicted in terms of their frequencies with 95% confidence interval. Frequencies were compared by Chi-square test or Fisher’s test and those with statistical significance (defined by *p* < 0.05) were included in a binary logistic regression analysis. We categorized variable “Age” as either more or less than 40 years old, because we assume that people younger than 40 years were immunized by vaccination following the national calendar of vaccination system in our country (born after 1982). Variables associated in the univariate analysis were included in a logistic regression multivariate analysis, adjusted by age and gender. All data analyses were performed using the SPSS statistical software package release 22·0 (IBM, Armonk, New York, United States).

### Ethical aspects

This study was designed and performed according to the Helsinki Declaration. The Andalusian Ethical Committee approved the study protocol. Samples were collected and cryopreserved at −80°C in the Andalusian Health System Biobank (National Register Reference: B.0001601).

## Findings

### Population

A total of 325 participants were enrolled in the study and underwent hepatitis B screening. Two hundred and eleven participants were not immunized against HBV (65%) and were considered candidates for immunization. We considered non-immunized those who presented anti-HBs concentration less than 10mIU/mL. No cases of active hepatitis B were detected. Baseline characteristic between immune and non-immune individuals is shown in Table 1. The median age of participants was 46 years old (38–54 Q1–Q3). The immunization rate among individuals younger than 40 years was higher than among older people (54.9% vs. 27.4%; *p* ≤ 0.001). Following multivariate analysis adjusting for gender, age emerged as the sole variable that achieved statistical significance (adjusted HR 2.96, CI95% 1.77 to 4.95, *p* < 0.001). None of the drugs were associated with immunization status.

**Table 1:** Baseline characteristics of participants according to their immune status against hepatitis B.

Variable	Category	Global 325 (100%)	No immune 211 (64.9%)	Immune 114 (35.1%)	*p*	HR	*P adjust*	*HR adjust*
Sex	Male	277 (85.2)	179 (64.6)	98 (35.4)	0.78	1.1 (0.5–2.1)	0.78	0.91 (0.46–1.79)
	Female	48 (14.8)	32 (66.7)	16 (33.3)		1		1
Age	<40 years	91 (28)	41 (45.1)	50 (54.9)	<0.001	0.3 (0.2–0.5)	<0.001	2.96 (1.77–4.95)
	≥40 years	234 (72)	170 (72.6)	64 (27.4)		1		1
**Drug use**								
Tobacco	Yes	109 (33.5)	69 (63.3)	40 (36.7)	0.66	1.1 (0.7–1.8)		
	No	216 (66.5)	142 (65.7)	74 (34.3)		1		
Synthesis drug	Yes	15 (4.6)	11 (73.3)	4 (26.7)	0.48	0.6 (0.2–2.1)		
	No	310 (95.4)	200 (64.5)	110 (35.5)		1		
Cocaine	Yes	177 (54.5)	110 (62.1)	67 (37.9)	0,25	1.3 (0.8–2.1)		
	No	140 (45.5)	101 (68.2)	47 (31.8)		1		
OST	Yes	72 (22.2)	44 (61.1)	28 (38.9)	0.44	1.2 (0.7–2.1)		
	No	253 (77.8)	167 (66)	86 (34)		1		
Alcohol	Yes	265 (81.5)	173 (65.3)	92 (34.7)	0.77	0.9 (0.5–1.6)		
	No	60 (18.5)	38 (63.3)	22 (36.7)		1		
Cannabis	Yes	130 (40)	73 (56.2)	57 (43.8)	0.007	1.9 (1.2–3.1)		1
	No	195 (60)	138 (70.8)	57 (29.2)		1	0.07	0.64 (0.39–1.04)

### Hepatitis B and SARS-CoV2 vaccination rate

From June 2020 to January 2021, there were 211 candidates for immunization to HBV and SARS-CoV2. Of them, only 15 participants received at least one dose of the hepatitis B vaccine (7.2%), and 9 of them were fully vaccinated (4.5%). No differences were found between the variables studied and the individuals who received vaccination or not (Table 2).

**Table 2:** Characteristics of participants according to vaccination against hepatitis B during the follow-up.

Variable	Category	Global *N* = 211 (100%)	Unvaccinated HBV *N* = 196 (92.8)	Vaccinated HBV *N* = 15 (7.2)	*p*	HR	*P adjust*	*HR adjust*
Sex	Male	179 (84.8)	168 (93.9)	11 (6.1)	0.25	0.46 (0.13–1.54)	0.18	0.43 (0.12–1.48)
	Female	32 (15.2)	28 (87.5)	4 (12.5)		1		1
Age	<40 years old	41 (19.4)	39 (95.1)	2 (4.9)	0.74	1.61 (0.35–7.45)	0.65	1
	≥40 years old	170 (80.6)	157 (92.4)	13 (7.6)		1		1.4 (0.3–6.7)
**Drug use**								
Tobacco	Yes	69 (32.7)	66 (95.7)	3 (4.3)	0.39	0.42 (0.13–1.8)		
	No	142 (67.3)	130 (91.5)	12 (8.5)		1		
Synthesis drugs	Yes	11 (5.2)	10 (90.9)	1 (9.1)	0.56	1.3 (0.16–11.14)		
	No	200 (94.8)	186 (93)	14 (7)		1		
Cocaine	Yes	110 (52.1)	102 (92.7)	8 (7.3)	0.99	1.05 (0.37–3,02)		
	No	101 (47.9)	94 (93.1)	7 (6.9)		1		
OST	Yes	44 (20.9)	41 (93.2)	3 (6.8)	0.99	0.94 (0.25–3.5)		
	No	167 (79.1)	155 (92.8)	12 (7.2)		1		
Alcohol	Yes	173 (82)	158 (91.3)	15 (8.7)	0.078	1.09 (1.05–1.15)	0.99	1
	No	38 (18)	38 (100)	0		1		1
Cannabis	Yes	73 (34.6)	68 (93.2)	5 (6.8)	0.99	0.94 (0.31–2.87)		
	No	138 (65.4)	128 (92.8)	10 (7.2)		1		
**SARS-Cov-2 vaccine**								
Vaccinated 1 doses	Yes	186 (88.15)	172 (92.5)	14 (7.5)	0.99	1.95 (0.25–15.5)		
	No	25 (11.85)	24 (96)	1 (4)				
Vaccinated > 2 doses	Yes	176 (83.41)	162 (92)	14 (8)	0.47	2.94 (0.37–23.1)		
	No	35 (16.6)	34 (97.1)	1 (2.9)				

During the same period, 186 participants (88.2%) received at least one dose of SARS-Cov-2 vaccine, of whom 176 were fully vaccinated (83.4%). Significant differences in vaccination rates were observed between HBV and SARS-CoV-2 vaccines recipients, both for those receiving at least one dose (7.2% vs. 88.2%; *p* < 0.001) and those who were fully vaccinated (4.5% vs. 83.42%; *p* < 0.001).

## Discussion

Vaccination against HBV should be a prioritized to eradicate this viral hepatitis and fulfill the WHO objective stablished for 2030 (13). In this context, the WHO the prioritization of hepatitis B vaccination in populations at high risk of infection. Among them, DAC-users constitute a difficult to access population. Different strategies have been tested to achieve vaccination of this group. The admission of DAC-users in closed institutions such detoxification centers represent a great opportunity to complete vaccination. Thus, in a study conducted in patients admitted to an opiate detoxification clinic, an 82% HBV vaccination rate was achieved with an accelerated vaccination schedule (14). This strategy would not be applicable to most DAC-users and, moreover, would only be effective in admissions so prolonged that vaccination schedules could be completed. Loss of follow-up of patients after discharge from a closed institution is common among DAC-users and is a major barrier to HBV vaccination strategies (15). In a study conducted in Brazil, among 553 crack users institutionalized in DAC, only 22% of patients completed a 21-day accelerated vaccination schedule. The main reason for such a small percentage was loss of follow-up of patients after discharge (median hospitalization time, 15 days) (16). Finally, several studies have suggested that strategies involving financial incentives for patients can improve vaccination rates in CAD users, although this cannot be extrapolated to other settings (15, 17, 18). In our study, after two-year follow-up, only 7.6% of those unimmunized against hepatitis B received at least one dose of vaccine. These results illustrate the difficulties to achieve high vaccination rates in DAC-users.

It has been observed that in situations where patients are connected to the health system, vaccination rates increase as the follow-up time is longer. Thus, in a cohort of HIV patients with 3 years of follow-up, hepatitis B vaccination rates of 9.6% were achieved (19), and in others with 7 and 10 years of follow-up, rates of 31.4% and 61.9%, were achieved, respectively (20, 21). In this context, after 20 years of follow-up, rates of 75.2% were obtained in a Needle Exchange Program (NEP) cohort (22). In any case, the vaccination rates obtained are far from optimal. Given the high risk of acquiring the infection and transmitting it to their environment among DAC-users, the objective should be to vaccinate the entire non-immunized population in the shortest period possible. All this suggests that one of the main reasons for vaccination failure in DAC-users is loss to follow-up and inability to contact patients after the loss of contact with the health care setting. However, attributing exclusively to this reason the responsibility for the failure of HBV vaccination strategies in CAD-users may be erroneous and constitute an obstacle to the design of strategies that accurately address the problem. In our experience, resources focused to support and treat substance disorders have limitations in collaborating with the Health System the follow-up of their users. These programs are external and founded by other associations or institutions. This situation complicates to identify risk factors for the healthcare providers and hinders the ability to screen or offer preventive measures for substance use disorders and Addiction Centers. It is strinking that the 88% of the DAC users included in our study received at least one dose of SARS-Cov-2 vaccine (83% completed vaccination) in the same observation time. This rate is comparable to those observed in the general population in Spain (23, 24). This fact suggests that it is possible to achieve high vaccination rates in DAC-users if appropriate strategies are employed.

The COVID-19 pandemic outbreak had an unexpected impact affecting all levels of the population worldwide. The rapid spread of the infection, due to transmission by air through Flügge droplets at close proximity (1 m) and Wells nuclei forming aerosols over longer distances, coupled with the elevated mortality rates observed during the two first waves (as of November 2023, 771,820,937 confirmed cases of COVID-19 and 6,978,175 reported deaths to WHO) (23), has, in our opinion, played a significant role in fostering widespread acceptance of vaccination among the general population. This way of transmission contrasts with hepatitis B transmission, which is predominantly by unprotected sexual intercourse and blood transmission (sharing syringes is a high risk in people with a history of drug abuse). We believe that these differences in risk perception could alter the population’s interest in protecting themselves from hepatitis B, even in the face of the high morbidity and mortality associated with chronic infection. Meanwhile, the measures implemented by governments to curb the outbreak, which originated in China and were subsequently adopted globally following the WHO’s declaration of a global health alert (including social restrictions and quarantine policies), had a profound impact on the population. These measures directly affected people’s lives, as they realized that taking precautions for their protection (such as using masks, adhering to hygiene measures, and getting vaccinated) had both social and economic repercussions for the general population and themselves. In Spain, the strategy to achieve high vaccination rates against SARS-Cov-2 included two essential elements: raising public awareness of the benefits of vaccination and facilitating access to vaccination. Achieving adequate awareness of the benefits of HBV vaccination in unimmunized DAC-users is an essential element in achieving high vaccination rates in this population, and to achieve this objective it is necessary to ensure that the health caregivers of the DAC-users themselves are also aware of it. Our findings suggest that clinicians should make a special effort to encourage HBV-unvaccinated patients to get vaccinated against HBV.

Our study has several limitations. It is retrospective with the limitations that this implies. The population studied is small, so our results should be interpreted with caution. Finally, our study was conducted in an area of universal and free health care, so our results may not necessarily be extrapolable to other populations with different socio-health care characteristics.

In conclusion, we found a high percentage of DACs-user unimmunized against HBV and the vaccination rates observed at 2 years was very low. This is a troubling finding that may compromise the goal of elimination of HBV infection proposed by WHO. The low vaccination rates against HBV contrast with the high vaccination rates against SARS-Cov-2 in the same population and at the same observation time, denoting that it is possible to achieve high vaccination rates in this population. Consequently, a similar approach for vaccination intended for SARS-CoV2 should be applied in high-risk population to warrant the success of immunization program against other preventable diseases such as HBV.

## Data availability statement

The raw data supporting the conclusions of this article will be made available by the authors, without undue reservation.

## Ethics statement

The studies involving humans were approved by Comité de Ética para la Investigación Biomédica de Andalucía (CEIC)—Nodo Córdoba. The studies were conducted in accordance with the local legislation and institutional requirements. The participants provided their written informed consent to participate in this study.

## Author contributions

DC-M: Data curation, Formal analysis, Investigation, Methodology, Writing – original draft, Writing – review & editing. AR-J: Formal analysis, Investigation, Methodology, Project administration, Resources, Supervision, Writing – original draft, Writing – review & editing. ÁC: Data curation, Investigation, Methodology, Writing – review & editing. LR-T: Data curation, Writing – review & editing. IR-C: Data curation, Writing – review & editing. AP: Writing – review & editing. BF: Writing – review & editing. DC-A: Writing – review & editing. MGC-G: Writing – review & editing. MVC-G: Writing – review & editing. JR-É: Writing – review & editing. AA-A: Writing – review & editing. LM-L: Writing – review & editing. LC: Writing – review & editing. IP-V: Investigation, Writing – original draft, Writing – review & editing, Data curation. AR: Funding acquisition, Investigation, Methodology, Project administration, Resources, Supervision, Validation, Writing – original draft, Writing – review & editing. ML-B: Writing – review & editing.

## References

[1] WHO. Hepatitis B. (2023). Available at: https://www.who.int/es/news-room/fact-sheets/detail/hepatitis-b

[2] Razavi-Shearer D Gamkrelidze I Nguyen MH Chen DS Van Damme P Abbas Z. Global prevalence, treatment, and prevention of hepatitis B virus infection in 2016: a modelling study. Lancet Gastroenterol Hepatol. (2018) 3:383–403. doi: 10.1016/S2468-1253(18)30056-6 29599078

[3] Pattyn J Hendrickx G Vorsters A Van Damme P Vaccines HB. Hepatitis B Vaccines. J Infect Dis. (2021) 224:S343–51. doi: 10.1093/infdis/jiaa668 34590138 PMC8482019

[4] Immunization Coverage. (2023). Available at: https://www.who.int/news-room/fact-sheets/detail/immunization-coverage

[5] Locarnini S Hatzakis A Chen DS Lok A. Strategies to control hepatitis B: public policy, epidemiology, vaccine and drugs. J Hepatol. (2015) 62:S76–86. doi: 10.1016/j.jhep.2015.01.018, PMID: 25920093

[6] Cui F Blach S Manzengo Mingiedi C Gonzalez MA Sabry Alaama A Mozalevskis A. Global reporting of progress towards elimination of hepatitis B and hepatitis C. Lancet Gastroenterol Hepatol. (2023) 8:332–42. doi: 10.1016/S2468-1253(22)00386-7, PMID: 36764320

[7] Degenhardt L Peacock A Colledge S Leung J Grebely J Vickerman P. Global prevalence of injecting drug use and sociodemographic characteristics and prevalence of HIV, HBV, and HCV in people who inject drugs: a multistage systematic review. Lancet Glob Health. (2017) e1192–e1207. 29074409 10.1016/S2214-109X(17)30375-3 PMC5683738

[8] Degenhardt L Peacock A Colledge S Leung J Grebely J Vickerman P. Global prevalence of injecting drug use and sociodemographic characteristics and prevalence of HIV, HBV, and HCV in people who inject drugs: a multistage systematic review. Lancet Glob Health. (2017) 5:e1192–207. doi: 10.1016/S2214-109X(17)30375-3, PMID: 29074409 PMC5683738

[9] Bowman S Grau LE Singer M Scott G Heimer R. Factors associated with hepatitis B vaccine series completion in a randomized trial for injection drug users reached through syringe exchange programs in three US cities. BMC Public Health. (2014) 14:820. doi: 10.1186/1471-2458-14-820 25107530 PMC4138371

[10] Corona-Mata D Rivero-Juárez A Camacho Á Ruiz-Torres L Ruiz-Cáceres I de la Fuente DB. Efficacy of a comprehensive strategy for the detection and treatment of hepatitis C infection in a population attending addiction centers. Front Public Health. (2023) 11:198. doi: 10.3389/fpubh.2023.1092960 PMC9932806 36817894

[11] de la Innovación A. Salud Y. Familias C., Por Jose Maria Torres Medina Fecha F DE. PROGRAMA DE VACUNACIÓN FRENTE A HEPATITIS A Y *B. arena* 1 Apdo Correos. (2019);17:41080.

[12] Estrategia de vacunación frente a COVID-19 en España, Actualización 11 del 8 de febrero de (2022). Available at: https://www.sanidad.gob.es/profesionales/saludPublica/prevPromocion/vacunaciones/covid19/Actualizaciones_Estrategia_Vacunacion/docs/COVID-19_Actualizacion11_EstrategiaVacunacion.pdf (Accessed May 23, 2023).

[13] WHO. Elimination of Hepatitis by 2030. (2023). Available at: https://www.who.int/healthtopics/hepatitis/elimination-of-hepatitis-by-2030#tab=tab_1 (Accessed 22 May 2023).

[14] Ramasamy P Lintzeris N Sutton Y Taylor H Day CA Haber PS. The outcome of a rapid hepatitis B vaccination programme in a methadone treatment clinic. Addiction. (2010) 105:329–34. doi: 10.1111/j.1360-0443.2009.02765.x 20078489

[15] Bridges CB Watson TL Nelson NP Chavez-Torres M Fineis P Ntiri-Reid B. Challenges with hepatitis B vaccination of high risk adults—a pilot program. Vaccine. (2019) 37:5111–20. doi: 10.1016/j.vaccine.2019.05.089, PMID: 31303523 PMC8819536

[16] da Silva LN da Silva França DD Del-Rio NHA dos Santos Carneiro MA Martins RMB Guimarães RA. Low prevalence, low immunization and low adherence to full hepatitis B vaccine scheme and high-risk behaviors among crack cocaine users in Central Brazil. J Infect Public Health. (2017) 10:76–83. doi: 10.1016/j.jiph.2016.02.010, PMID: 27026240

[17] Topp L Day CA Wand H Deacon RM van Beek I Haber PS. A randomised controlled trial of financial incentives to increase hepatitis B vaccination completion among people who inject drugs in Australia. Prev Med. (2013) 57:297–303. doi: 10.1016/j.ypmed.2013.04.013 23639625

[18] Seal KH Kral AH Lorvick J McNees A Gee L Edlin BR. A randomized controlled trial of monetary incentives vs. outreach to enhance adherence to the hepatitis B vaccine series among injection drug users. Drug Alcohol Depend. (2003) 71:127–31. doi: 10.1016/S0376-8716(03)00074-7 12927650

[19] Weiser J Perez A Bradley H King H Shouse RL. Low prevalence of Hepatitis B vaccination among patients receiving medical care for HIV infection in the United States, 2009 to 2012. Ann Intern Med. (2018) 168:245–54. doi: 10.7326/M17-1689, PMID: 29277848 PMC5820114

[20] Bailey CL Smith V Sands M. Hepatitis B vaccine: a seven-year study of adherence to the immunization guidelines and efficacy in HIV-1-positive adults. Int J Infect Dis. (2008) 12:e77–83. doi: 10.1016/j.ijid.2008.05.1226 18723381

[21] Valour F Cotte L Voirin N Godinot M Ader F Ferry T. Vaccination coverage against hepatitis a and B viruses, *Streptococcus pneumoniae*, seasonal flu, and a(H1N1)2009 pandemic influenza in HIV-infected patients. Vaccine. (2014) 32:4558–64. doi: 10.1016/j.vaccine.2014.06.015 24951870

[22] Alanko Blomé M Björkman P Flamholc L Jacobsson H Widell A. Vaccination against hepatitis B virus among people who inject drugs—a 20year experience from a Swedish needle exchange program. Vaccine. (2017) 35:84–90. doi: 10.1016/j.vaccine.2016.11.041, PMID: 27894721

[23] WHO Coronavirus (COVID-19) Dashboard WHO coronavirus (COVID-19) dashboard with vaccination data. (2023). Available at: https://covid19.who.int/?mapFilter=cases

[24] Ministerio de Sanidad Profesionales—Cuadro de mando resumen de datos de vacunación. (2023). Available at: https://www.sanidad.gob.es/areas/alertasEmergenciasSanitarias/alertasActuales/nCov/pbiVacunacion.htm

